# Differential Effects of Comorbidity on Antihypertensive and Glucose-Regulating Treatment in Diabetes Mellitus – A Cohort Study

**DOI:** 10.1371/journal.pone.0038707

**Published:** 2012-06-05

**Authors:** Jaco Voorham, Flora M. Haaijer-Ruskamp, Bruce H. R. Wolffenbuttel, Dick de Zeeuw, Ronald P. Stolk, Petra Denig

**Affiliations:** 1 Department of Clinical Pharmacology, Faculty of Medical Science, University Medical Center Groningen, University of Groningen, Groningen, The Netherlands; 2 Department of Epidemiology, Faculty of Medical Science, University Medical Center Groningen, University of Groningen, Groningen, The Netherlands; 3 Graduate School for Health Research, University Medical Center Groningen, University of Groningen, Groningen, The Netherlands; 4 Department of Endocrinology, Faculty of Medical Science, University Medical Center Groningen, University of Groningen, Groningen, The Netherlands; Fundación para la Prevención y el Control de las Enfermedades Crónicas No Transmisibles en América Latina (FunPRECAL), Argentina

## Abstract

**Background:**

Comorbidity is often mentioned as interfering with “optimal” treatment decisions in diabetes care. It is suggested that diabetes-related comorbidity will increase adequate treatment, whereas diabetes-unrelated comorbidity may decrease this process of care. We hypothesized that these effects differ according to expected priority of the conditions.

**Methods:**

We evaluated the relationship between comorbidity and treatment intensification in a study of 11,248 type 2 diabetes patients using the GIANTT (Groningen Initiative to Analyse type 2 diabetes Treatment) database. We formed a cohort of patients with a systolic blood pressure ≥140 mmHg (6,820 hypertensive diabetics), and a cohort of patients with an HbA1c ≥7% (3,589 hyperglycemic diabetics) in 2007. We differentiated comorbidity by diabetes-related or unrelated conditions and by priority. High priority conditions include conditions that are life-interfering, incident or requiring new medication treatment. We performed Cox regression analyses to assess association with treatment intensification, defined as dose increase, start, or addition of drugs.

**Results:**

In both the hypertensive and hyperglycemic cohort, only patients with incident diabetes-related comorbidity had a higher chance of treatment intensification (HR 4.48, 2.33–8.62 (p<0.001) for hypertensives; HR 2.37, 1.09–5.17 (p = 0.030) for hyperglycemics). Intensification of hypertension treatment was less likely when a new glucose-regulating drug was prescribed (HR 0.24, 0.06–0.97 (p = 0.046)). None of the prevalent or unrelated comorbidity was significantly associated with treatment intensification.

**Conclusions:**

Diabetes-related comorbidity induced better risk factor treatment only for incident cases, implying that appropriate care is provided more often when complications occur. Diabetes-unrelated comorbidity did not affect hypertension or hyperglycemia management, even when it was incident or life-interfering. Thus, the observed “undertreatment” in diabetes care cannot be explained by constraints caused by such comorbidity.

## Introduction

Although considerable progress has been achieved in the quality of diabetes care, there remains a gap between what treatment guidelines indicate as appropriate care and what is observed in practice [1], [2]. There is concern that comorbidities may affect the quality of care, and interfere with adequate treatment in insufficiently controlled patients [3]. Multimorbidity may result in trade-off decisions and prioritization regarding therapeutic management [4], [5]. Treatment complexity in patients with comorbidity and competing demands are reported as reasons for not acting as recommended by treatment guidelines [5]–[7]. Competing demands during encounters require that patients and clinicians prioritize what is to be done, and may defer some actions to subsequent visits [8]–[10]. Comorbidity and incompatible treatment plans may require trade-offs that will result in justified noncompliance with guidelines [11]–[14].

The interaction between comorbidity and treatment of cardiovascular risk factors in patients with diabetes is only partly understood [3], [4], [15], [16]. Comorbidity may increase as well as decrease the chance of treatment intensification in insufficiently controlled patients [8], [15]–[22]. To understand these effects better, the type of the comorbid condition should be taken into account [4], [16], [20]. It has been suggested that diabetes-related comorbidity enhances cardiovascular risk factor management but this does not seem to be the case always [4], [16], [17]. Unrelated comorbidity may have no impact or have a negative effect on risk factor management [15], [16]. These inconsistent effects could be due to differences in clinical dominance or priority of the comorbid condition in comparison to the target condition, *i.e.* hypertension or hyperglycemia. Different priorities could be expected for incident vs. prevalent comorbidity, acute vs. chronic conditions, and somatic vs. psychiatric or malignant conditions [3], [10], [17].

We examined the impact of the different types of comorbidity on the decision to intensify medication treatment for two common conditions in patients with diabetes: (1) hypertension, and (2) hyperglycemia.

## Methods

### Design and data collection

We conducted two cohort studies to evaluate the association between comorbidity and medication treatment decisions for two conditions in patients with diabetes. Prescriptions, clinical measurements, comorbidity and demographic data for patients with type 2 diabetes were collected from the Groningen Initiative to Analyse Type 2 diabetes Treatment (GIANTT) database. This database contains anonymized longitudinal information retrieved from electronic medical records (EMR) of general practitioners (GPs). The GPs prescribe electronically using the EMR system, ensuring full information on prescribed drugs and dosing schemes. Clinical measurements are collected using a validated computerized extraction method, ensuring that all relevant data from the medical records are included [23]. Data on comorbid conditions are collected from the problem or episode lists in the EMR where the GPs can document symptoms, diagnoses, and (surgical) interventions using either the International Classification for Primary Care version 1 (ICPC) [24] coding or text lines, which were recoded into the corresponding ICPC codes or additional codes for relevant procedures. Diabetes duration was calculated using the date of diagnosis as provided by the responsible GP. In The Netherlands, patients are registered with a single GP who acts as a gatekeeper and is responsible for maintaining the patient's medical record.

According to the Code of Conduct for Health Research (Dutch FMWV Code approved by Dutch Data Protection Authority in 2004), no ethics committee approval is needed for research using anonymous medical records and does not lead to questions from the point of view of privacy.

### Study population

**Figure 1:**
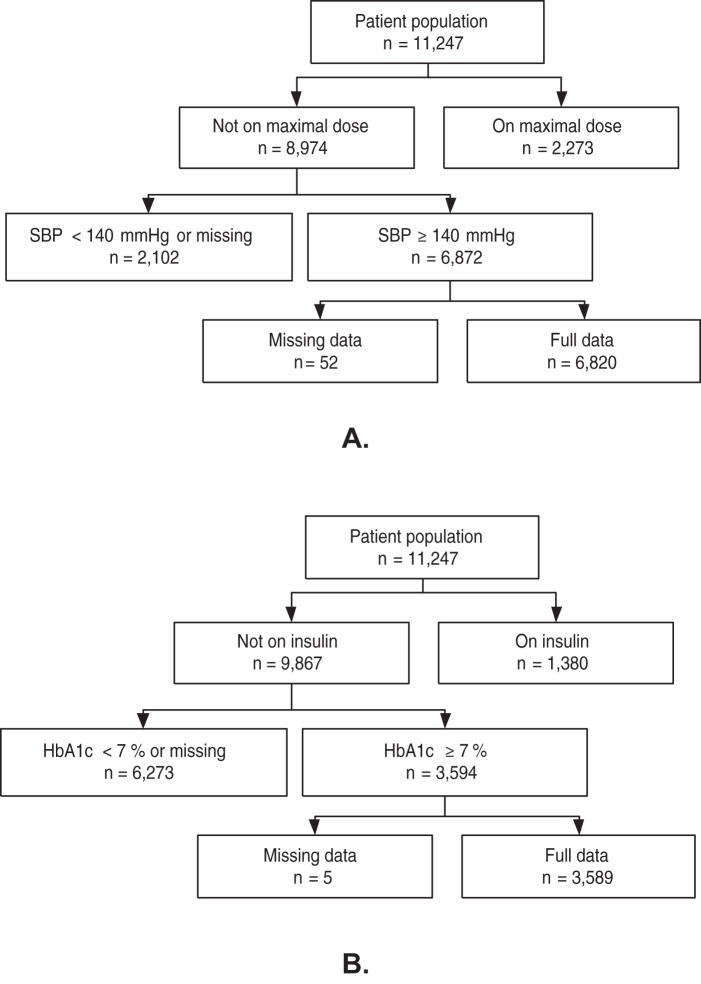
Patient selection for hypertensive (A) and hyperglycemia (B) study cohort.

The study population consists of all patients managed by 142 GPs from the northern Netherlands with a GP confirmed diagnosis of type 2 diabetes mellitus on January 1^st^ 2007, and present in the practice throughout 2007. Two cohorts of diabetes patients were formed with a risk factor measurement above treatment target in 2007: 1. Systolic blood pressure (SBP) ≥140 mmHg (hypertensive cohort), 2. HbA1c ≥7% (hyperglycemia cohort), excluding patients already receiving maximal treatment in whom further intensification by the GP is not indicated or with incomplete prescription data (Figure 1). Maximal treatment was defined as receiving already ≥3 antihypertensive drug classes on maximal maintenance dose for the hypertensive cohort, or receiving insulin for the hyperglycemia cohort. To be able to evaluate the impact of related comorbidity on the treatment decisions for hypertension and for hyperglycemia, patients with both conditions were included in both cohorts.

### Treatment decisions

We defined the index observation for hypertensive patients as the first observation of an elevated blood pressure level in 2007, and for hyperglycemic patients as the first observation of an elevated HbA1c level in 2007. Patients could contribute to both cohorts but not necessarily with the same index date. We assessed treatment changes during a 120-days follow-up period, if necessary using data from 2008, to capture actions that were postponed until the next regular control visit as suggested previously [25]. Treatment intensification is defined as any dose increase and/or addition or start of a new drug class. Patients with dosage modifications into opposite directions were excluded (*e.g.* a dose increase coinciding with a dose decrease in one or more drugs for the same indication) (n = 27 for hypertensive cohort, n = 33 for hyperglycemia cohort).

### Comorbidity and concomitant conditions

Based on the typology suggested by Piette & Kerr [4] and subsequent studies using this framework [10], [15], [16], [26], we distinguish two main dimensions with regard to the comorbid conditions, *i.e.* 1) concordance with diabetes, and 2) clinical priority. We classified diseases, conditions and symptoms (Table 1), and also new medication starts according to this typology:

**Table 1:** Classification scheme for comorbidity typology.

	Diabetes-unrelated	Diabetes-related	Priority
*Acute, intermittent or recurring conditions*	Infections, injuries, inflammatory diseases, allergies, benign neoplasms, neurological problems, skin problems, eye and ear problems	Myocardial infarction, stroke, diabetic neuropathy, proteinuria, nephropathy, retinopathy, low extremity ulcers & amputations, coronary and peripheral vascular interventions	May require immediate attention
*Chronic somatic conditions*	Asthma, COPD, rheumatoid artritis, arthrosis, osteoporosis, chronic back syndromes, chronic gastrointestinal diseases, disabilities	Coronary and peripheral vascular diseases*, heart failure, arrythmias, lipid disorders, endstage renal disease, blindness	May require immediate attention when incident; no clear priority when prevalent
*Psychiatric conditions*	All psychiatric disorders	–	May be dominant
*Malignant conditions*	All malignant neoplasms	–	May be dominant

* hypertension included only for hyperglycemic management.

1. Concordance to diabetes: related versus unrelated conditions.
2. Degree of priority.

- Acute or incident conditions which may require immediate but transitory attention that may delay hypertension or hyperglycemia management.
- Somatic conditions for which the dominance is not clear.
- Major life-interfering conditions (psychiatric, malignant), that may be dominant over hypertension or hyperglycemia management.
- New medication starts, that may compete with simultaneous indicated treatment changes for hypertension or hyperglycemia.

Diabetes-related conditions represent part of the same overall (pathophysiologic) risk profile, and are more likely to be the focus of the same disease and management plan [4]. All diagnosis codes of the ICPC-1 list were classified by two general practitioners as either chronic or acute conditions, the latter including also recurring and intermittent conditions. Chronic conditions documented prior to the index date were included as prevalent conditions. A new medication start was defined as a prescription for a drug identified at the lowest ATC-level [27] that had not been prescribed in the previous 180 days. Diabetes-related drugs were subdivided into antihypertensive, glucose-regulating, lipid-regulating drugs and aspirin.

### Statistical analysis

We conducted separate analyses to study treatment intensification for each condition using multivariate Cox Proportional Hazard regression with shared frailty to correct for clustering at practitioner level (STATA version 11). Patients were censored at 120 days if no intensification occurred. The risk factor levels, that is, systolic blood pressure measurements for the hypertensive cohort, and HbA1c measurements for the hyperglycemic cohort, were added to each model as a time-dependent covariate. Since a patient may be both hypertensive and hyperglycemic, concurrent related conditions and treatment decisions were included in the models. Prevalent chronic conditions were added to the models as fixed variables. Presence of any psychiatric disease or malignancy in a 10-year period prior to the index date were each included as a binary variable. Other chronic somatic diseases were included as the total number of distinct conditions. The effect of incident life-interfering conditions started on the day of their registration in the patient record and lasted until the end of follow-up. All other acute or incident conditions and new medication starts were added to the models as time-dependent variables with a limited effect duration of 7 days, since their impact is assumed to be transitory. Sensitivity analysis was performed using a period of 14 days to assess the impact of this model assumption.

The following potential confounders were added to the models as fixed covariates scored at the index date: age, diabetes duration, sex, and medication use at baseline, *i.e.* polypharmacy (≥4 other chronic drugs), and whether the patient was already using antihypertensive or glucose-regulating drugs during a 6-months period prior to the index date.

To establish possible differences in the associations studied between people already on medication and new users, we explored interactions between use of the antihypertensive and glucose-regulating medication at baseline and the explanatory variables, *e.g.* the comorbidity conditions and new medication starts. Since none of the interaction terms were found to contribute significantly to the models, we included medication use at baseline only as a covariate.

We conducted another sensitivity analysis to assess the effect of limited comorbidity registration at GP level on the models. For this, we calculated the average number of documented comorbidities per patient at GP level and repeated the modeling excluding GPs in the lowest tercile regarding this number. We performed explorative analyses using subclasses of comorbidity within the ones defined by our typology at the level of ICPC chapters and components, to verify that the defined categories were not composed of subclasses with opposite effects on the outcome. We found no indications for this.

Results are presented as Hazard Ratios with 95% confidence intervals.

## Results

Of the 11,248 patients in the study population, respectively 6,820 and 3,589 were included in hypertension and hyperglycemia cohorts (Table 2). The rate of treatment intensification was higher in glycemic management (38.1%) compared to hypertension management (16.4%). The comorbidity and concomitant conditions are described in Table 3, by cohort and intensification status. It reports the number of patients with at least 1 occurrence of a condition during follow-up.

**Table 2:** Characteristics of the hypertensive and the hyperglycemia cohorts.

	Hypertensive	Hyperglycemia
Number of patients	6,820	3,589
Untreated (% of total)	1,652 (24.2%)	514 (14.3%)
Started (% of untreated)	329 (19.9%)	274 (53.3%)
Treated (% of total)	5,168 (75.8%)	3,075 (85.7%)
Intensified (% of treated)	788 (15.2%)	1,093 (35.5%)
Female, percentage	54.4 %	50.4 %
Systolic blood pressure in mmHg, mean (SD)	154 (15)	142 (20)
HbA1c in %, mean (SD)	6.8 (1.0)	7.6 (0.9)
Age in years, mean (SD)	68 (12)	66 (12)
Diabetes duration in years, median (IQR)	5 (7)	5 (6.5)
Polypharmacy (≥4 drugs)	56.2%	55.8%

SD  =  standard deviation, IQR  =  interquartile range.

**Table 3:** Overview of presence of comorbidity, concomitant conditions and events, N (%).

			Hypertensive		Hyperglycemia	
			Not intensified (n = 5,703)	Intensified (n = 1,117)	Not intensified (n = 2,222)	Intensified (n = 1,367)
Incident ≥1	Diabetes-related		174 (3.1)	37 (3.3)	67 (3.0)	23 (1.7)
	Unrelated	Somatic	632 (11.1)	71 (6.4)	254 (11.4)	58 (4.2)
		Psychiatric	16 (0.3)	3 (0.3)	4 (0.2)	2 (0.1)
		Malignancies	35 (0.6)	2 (0.2)	13 (0.6)	3 (0.2)
Prevalent ≥1	Diabetes-related		1684 (29.5)	346 (31.0)	1061 (47.7)	628 (45.9)
	Unrelated	Somatic	1375 (24.1)	300 (26.9)	511 (23.0)	328 (24.0)
		Psychiatric	227 (4.0)	42 (3.8)	87 (3.9)	58 (4.2)
		Malignancies	447 (7.8)	81 (7.3)	150 (6.8)	97 (7.1)
Drugs started ≥1	Diabetes-related	Antihypertensive	0 (0.0)	0 (0.0)	239 (10.8)	67 (4.9)
		Glucose-regulating	633 (11.1)	62 (5.6)	0 (0.0)	0 (0.0)
		Lipid-regulating	361 (6.3)	42 (3.8)	138 (6.2)	44 (3.2)
		ASA	87 (1.5)	20 (1.8)	38 (1.7)	8 (0.6)
	Unrelated		2863 (50.2)	292 (26.1)	1074 (48.3)	293 (21.4)

### Antihypertensive treatment

Incident diabetes-related comorbidity increased the chance of treatment intensification more than fourfold (Table 4, HR 4.48, 2.33–8.62 (p<0.001)). Treatment intensification was not affected by any incident unrelated comorbidity or any prevalent comorbidity. The start of a new glucose-regulating drug reduced the chance of treatment intensification for hypertension by 76% (HR 0.24, 0.06–0.97 (p = 0.046)). The start of other drugs did not affect the treatment decisions.

**Table 4:** Results of the Cox proportional hazard models for the hypertensive and hyperglycemia cohorts.

	Hypertensive cohort (n = 6,820)			Hyperglycemia cohort (n = 3,589)		
Factor	HR	P-value	95% CI	HR	P-value	95% CI
Incident diabetes-related	4.48	<0.001	2.33–8.62	2.37	0.030	1.09–5.17
Incident unrelated psychiatric	1.77	0.329	0.56–5.62	3.69	0.071	0.89–15.24
Incident unrelated malignant	0.90	0.877	0.22–3.61	0.90	0.854	0.28–2.83
Incident unrelated somatic	1.18	0.566	0.67–2.11	0.84	0.555	0.47–1.50
Prevalent diabetes-related	0.99	0.634	0.95–1.03	0.97	0.039	0.94–1.00
Prevalent unrelated psychiatric	0.96	0.786	0.70–1.31	1.08	0.575	0.82–1.42
Prevalent unrelated malignant	0.96	0.747	0.76–1.22	1.04	0.691	0.84–1.30
Prevalent unrelated somatic	1.00	0.885	0.96–1.05	1.03	0.182	0.99–1.07
New glucose-regulating drug started	0.24	0.046	0.06–0.97	NA		
New antihypertensive drug started	NA			0.50	0.072	0.24–1.06
New lipid-regulating drug started	1.49	0.339	0.66–3.36	0.91	0.794	0.43–1.91
Aspirin started	0.62	0.639	0.08–4.59	-	-	-
New unrelated drug started	1.01	0.927	0.75–1.37	0.87	0.314	0.67–1.14
Systolic blood-pressure (10 mmHg)	1.44	<0.001	1.40–1.48	NA		
HbA1c (1%)	NA			1.34	<0.001	1.28–1.40
Age (10 yrs)	0.95	0.076	0.90–1.01	0.96	0.087	0.92–1.01
Female	0.93	0.261	0.83–1.05	1.02	0.763	0.91–1.13
Diabetes duration (10 yrs)	0.87	0.012	0.78–0.97	0.59	<0.001	0.52–0.67
Polypharmacy (≥4 drugs)	1.16	0.023	1.02–1.31	1.00	0.974	0.89–1.12
Current antihypertensive drug user	0.65	<0.001	0.56–0.74	NA		
Current oral antidiabetic drug user	NA			0.59	<0.001	0.52–0.68

HR  =  Hazard ratio; P  =  probability; 95% CI = 95% confidence interval; NA  =  not applicable.

### Glucose-regulating treatment

Incident diabetes-related comorbidity increased the chance of glucose-regulating treatment intensification (Table 4, HR 2.37, 1.09–5.17 (p = 0.030)). Incident unrelated comorbidity did not affect these treatment decisions. Also, the start of other drugs was not significantly associated with this treatment intensification. Prevalent diabetes-related comorbidity reduced the chance of such treatment changes by 3% (HR 0.97, 0.94–1.00 (p = 0.039)).

### Impact of medication use at baseline

The chance to intensify medication was lower for patients already on medication treatment in comparison to patients not yet receiving medication (Table 4, HR 0.65, 0.56–0.74 (p<0.001) and HR 0.59, 0.52–0.68 (p<0.001) for current users of antihypertensive and glucose-regulating medication). Polypharmacy (concurrent use of ≥4 chronic drugs) did not affect the treatment intensification in the hyperglycemia cohort but was positively associated with treatment intensification in the hypertensive patients (Table 4).

### Sensitivity analyses

After excluding practices with the lowest number of comorbidity records per patient, the hazard ratios of the comorbidity variables did not change meaningfully (tables S1 and S2). The effect of new glucose-regulating drug starts on treatment intensification, however, lost significance in the limited dataset. Using effect duration of 14 days instead of 7 days for incident conditions also did not result in meaningful changes in model results (tables S1 and S2).

## Discussion

Our study shows that new occurrences of diabetes-related conditions increased the chance of intensifying antihypertensive and glucose-regulating treatment in patients with elevated risk factor levels. We could not confirm that already existing diabetes-related conditions had a positive effect on treatment intensifications. Furthermore, we found no evidence that any diabetes-unrelated comorbidity competed with diabetes management, although we tried to classify the conditions according to their possible dominance. The only competing effect we observed was the negative effect of the start of a new glucose-regulating drug on intensifying antihypertensive treatment.

The distinction between diabetes-related and unrelated comorbidity is relevant for better understanding the impact of comorbidity on diabetes management. Our study confirms a recent finding that diabetes-related conditions increase the chance of appropriate follow-up risk factor treatment whereas unrelated conditions have no such effect [16]. Our finding that this is only the case for incident diabetes-related conditions, whereas no significant association is observed for prevalent diabetes-related conditions, suggests that the GPs wait too long before intensifying treatment, *i.e.* when complications occur.

The lack of a negative effect of diabetes-unrelated conditions seems to contradict the finding that discussing other conditions during a clinical encounter decreases the chance of an antihypertensive treatment change at a single visit [8], [28]. However, competition during a single visit clearly differs from the perspective of looking at appropriate follow-up care within a longer period, which was considered in our study as well as in the study of Woodard *et al*. [16] We also do not see any impact of prevalent comorbidity, regardless of its relation to diabetes or priority, on diabetes management. In other studies, conflicting results have been found regarding prevalent comorbidity. The number of diabetes-related conditions documented in the medical record was not associated with antihypertensive treatment intensification in one study [17]. In two other studies, however, lower as well as higher rates of treatment intensification were observed in diabetes patients with other chronic conditions [18], [28]. This lack of consistent findings could partly be caused by differences in definitions of the included comorbidities. We differentiated between diabetes-related and unrelated, as well as acute or incident, somatic, psychiatric and malignant conditions. Especially the somatic conditions may still include a mixture of highly relevant and less important conditions that could mask possible diverging effects. The lack of impact of psychiatric and malignant conditions, however, was unexpected given other findings [15]. Differences in patient population might play a role. Our study population consists of primary care patients with a mean age of 67 years and diabetes duration of 6 years who were not yet treated with insulin. Most other studies looking at the effect of unrelated comorbidity on diabetes management have included predominantly male veteran populations which were either substantially older, had a longer diabetes duration, or were treated by internists [8], [15], [18], [28]. It could be that other prevalent comorbidity becomes more dominant over diabetes management with increasing age.

Competition for time and priority may occur when multiple changes in drug treatment are indicated. Previously it was found that prescribing of medication for an acute condition was negatively associated with the intensification of antihypertensive medication in diabetes patients [17]. We observed a similar lower chance for antihypertensive treatment intensification when a new glucose-regulating drug was prescribed. We did not observe this kind of competition for other drug starts nor for glucose-regulating treatment intensification. This could be indicative of a higher priority that may be put on glycemic over hypertensive management. Indications for such prioritization have been reported before [2], [17], [29], [30]. This is worrisome since adequate hypertensive management is especially important to prevent cardiovascular and renal complications in this patient population.

### Strength and limitations

Our study is one of the first trying to disentangle the effects of comorbidity on treatment intensification by differentiating for the type and nature of different demands. We examined two conditions where some patients could contribute in both cohorts, but we expected no inflation of results since the inclusion criteria and the outcomes clearly differed. We include a follow-up period for appropriate care of 120 days to allow for actions that were postponed until the next regular control visit. In other health care settings, it might be appropriate to include a follow-up period of up to 6 months [16], [31], [32]. It can be expected that differences in health care organization influence chronic disease management. About half of the GPs in our study worked with a diabetes assistant or nurse practitioner to support diabetes care. Including the presence of diabetes support staff as a binary covariate in the models did not change the outcomes (results not shown). We included a large primary care population in contrast to previous studies that were often conducted in elderly veterans populations.

Most of the comorbidity and events were present in less than 10% of the patients, resulting in low precision effect estimates. Chance findings cannot be ruled out when including multiple factors. The directions and sizes of effects in the full and the limited models (tables S1 and S2) were similar, making it less likely to be chance findings. We used comorbidity as documented in the medical records. This can be an underestimation of all actual problems or events that may compete with chronic disease management. We have included the start of new medication as a clear action competing with care but we were not able to incorporate the effect of other interventions, such as discussion of life style issues or of medication adherence.

### Conclusion

In our cohort of Dutch primary care patients with diabetes, we did not find evidence that prevalent comorbidity competes with hypertension and hyperglycemia management when a follow-up period for appropriate management is included. This implies that the presence of comorbidity does not explain the low levels of treatment intensification observed. The higher level of treatment intensification in patients with incident diabetes-related conditions seems encouraging. However, given the observed “undertreatment” it may imply that appropriate care is partly provided at a late stage, namely when new complications occur.

## Supporting Information

Table S1 **Cox proportional hazard models for hypertensive cohort: sensitivity analysis excluding practices with lowest comorbidity records (limited data) and extending effect duration for incident events from 7 to 14 days.**
(DOC)

Table S2 **Cox proportional hazard models for hyperglycemic cohort: sensitivity analysis excluding practices with lowest comorbidity records (limited data) and extending effect duration for incident events from 7 to 14 days.**
(DOC)
