# Supplementary material for: Differential Effects of Comorbidity on Antihypertensive and Glucose-Regulating Treatment in Diabetes Mellitus – A Cohort Study
Source: PLoS One. 2012 Jun 5;7(6):e38707. doi: 10.1371/journal.pone.0038707 (PMC3367971; doi:10.1371/journal.pone.0038707)
Supplement: Table S1 — Cox proportional hazard models for hypertensive cohort: sensitivity analysis excluding practices with lowest comorbidity records (limited data) and extending effect duration for incident events from 7 to 14 days. (DOC) [file pone.0038707.s001.doc]

Table S1. Cox proportional hazard models for hypertensive cohort: sensitivity analysis excluding practices with lowest comorbidity records (limited data) and extending effect duration for incident events from 7 to 14 days.

|  | **7 days effect** | | | | | | **14 days effect** | | | | | |
| --- | --- | --- | --- | --- | --- | --- | --- | --- | --- | --- | --- | --- |
|  | **Full data (n=6,820)** | | | **Limited data (n=4,730)** | | | **Full data (n=6,820)** | | | **Limited data (n=4,730)** | | |
| Factor | **HR** | **P** | **95% CI** | **HR** | **P** | **95% CI** | **HR** | **P** | **95% CI** | **HR** | **P** | **95% CI** |
| Incident diabetes-related | 4.48 | 0.000 | 2.33-8.62 | 4.29 | 0.000 | 2.06-8.95 | 3.65 | 0.000 | 2.14-6.21 | 3.43 | 0.000 | 1.89-6.23 |
| Incident unrelated psychiatric | 1.77 | 0.329 | 0.56-5.62 | 1.30 | 0.714 | 0.32-5.30 | 1.71 | 0.364 | 0.54-5.41 | 1.25 | 0.752 | 0.31-5.12 |
| Incident unrelated malignant | 0.90 | 0.877 | 0.22-3.61 | 1.04 | 0.955 | 0.26-4.21 | 0.87 | 0.843 | 0.22-3.51 | 1.00 | 0.997 | 0.25-4.06 |
| Incident unrelated somatic | 1.18 | 0.566 | 0.67-2.11 | 1.10 | 0.770 | 0.57-2.13 | 1.24 | 0.338 | 0.80-1.91 | 1.21 | 0.428 | 0.75-1.96 |
| Prevalent diabetes-related | 0.99 | 0.634 | 0.95-1.03 | 0.98 | 0.529 | 0.94-1.03 | 0.99 | 0.611 | 0.95-1.03 | 0.98 | 0.514 | 0.94-1.03 |
| Prevalent unrelated psychiatric | 0.96 | 0.786 | 0.70-1.31 | 0.98 | 0.918 | 0.70-1.38 | 0.96 | 0.782 | 0.70-1.31 | 0.98 | 0.907 | 0.70-1.37 |
| Prevalent unrelated malignant | 0.96 | 0.747 | 0.76-1.22 | 0.91 | 0.461 | 0.70-1.17 | 0.96 | 0.709 | 0.75-1.21 | 0.90 | 0.445 | 0.70-1.17 |
| Prevalent unrelated somatic | 1.00 | 0.885 | 0.96-1.05 | 1.00 | 0.925 | 0.95-1.05 | 1.00 | 0.887 | 0.96-1.05 | 1.00 | 0.924 | 0.95-1.05 |
| New glucose-regulating drug started | 0.24 | 0.046 | 0.06-0.97 | 0.17 | 0.077 | 0.02-1.22 | 0.24 | 0.047 | 0.06-0.98 | 0.17 | 0.079 | 0.02-1.23 |
| New lipid-regulating drug started | 1.49 | 0.339 | 0.66-3.36 | 1.48 | 0.441 | 0.55-4.02 | 1.50 | 0.326 | 0.67-3.39 | 1.50 | 0.427 | 0.55-4.06 |
| Aspirin started | 0.62 | 0.639 | 0.08-4.59 | 0.97 | 0.976 | 0.13-7.34 | 0.64 | 0.662 | 0.09-4.71 | 1.01 | 0.990 | 0.14-7.59 |
| New unrelated drug started | 1.01 | 0.927 | 0.75-1.37 | 0.82 | 0.315 | 0.55-1.21 | 1.01 | 0.969 | 0.75-1.35 | 0.80 | 0.282 | 0.54-1.20 |
| Systolic blood-pressure (10 mmHg) | 1.44 | 0.000 | 1.40-1.48 | 1.44 | 0.000 | 1.39-1.49 | 1.44 | 0.000 | 1.40-1.48 | 1.44 | 0.000 | 1.39-1.49 |
| Age (10 yrs) | 0.95 | 0.076 | 0.90-1.01 | 0.96 | 0.185 | 0.89-1.02 | 0.95 | 0.075 | 0.90-1.01 | 0.96 | 0.183 | 0.89-1.02 |
| Female | 0.93 | 0.261 | 0.83-1.05 | 0.91 | 0.224 | 0.79-1.06 | 0.93 | 0.258 | 0.82-1.05 | 0.91 | 0.222 | 0.79-1.06 |
| Diabetes duration (10 yrs) | 0.87 | 0.012 | 0.78-0.97 | 0.90 | 0.073 | 0.80-1.01 | 0.87 | 0.011 | 0.78-0.97 | 0.90 | 0.072 | 0.80-1.01 |
| Polypharmacy (≥4 drugs) | 1.16 | 0.023 | 1.02-1.31 | 1.23 | 0.008 | 1.06-1.43 | 1.16 | 0.024 | 1.02-1.31 | 1.23 | 0.008 | 1.05-1.43 |
| Current antihypertensive drug user | 0.65 | 0.000 | 0.56-0.74 | 0.67 | 0.000 | 0.57-0.79 | 0.65 | 0.000 | 0.56-0.74 | 0.67 | 0.000 | 0.57-0.79 |
|  |  |  |  |  |  |  |  |  |  |  |  |  |

HR = Hazard ratio, P = P-value, CI = Confidence Interval
