# Supplementary material for: Differential Effects of Comorbidity on Antihypertensive and Glucose-Regulating Treatment in Diabetes Mellitus – A Cohort Study
Source: PLoS One. 2012 Jun 5;7(6):e38707. doi: 10.1371/journal.pone.0038707 (PMC3367971; doi:10.1371/journal.pone.0038707)
Supplement: Table S2 — Cox proportional hazard models for hyperglycemic cohort: sensitivity analysis excluding practices with lowest comorbidity records (limited data) and extending effect duration for incident events from 7 to 14 days. (DOC) [file pone.0038707.s002.doc]

Table S2. Cox proportional hazard models for hyperglycemic cohort: sensitivity analysis excluding practices with lowest comorbidity records (limited data) and extending effect duration for incident events from 7 to 14 days.

|  | **7 days effect** | | | | | | **14 days effect** | | | | | |
| --- | --- | --- | --- | --- | --- | --- | --- | --- | --- | --- | --- | --- |
|  | **Full data (n=3,589)** | | | **Limited data (n=2,386)** | | | **Full data (n=3,589)** | | | **Limited data (n=2,386)** | | |
| **Factor** | **HR** | **P** | **95% CI** | **HR** | **P** | **95% CI** | **HR** | **P** | **95% CI** | **HR** | **P** | **95% CI** |
| Incident diabetes-related | 2.37 | 0.030 | 1.09-5.17 | 2.55 | 0.020 | 1.16-5.61 | 1.95 | 0.045 | 1.02-3.75 | 2.30 | 0.013 | 1.19-4.44 |
| Incident unrelated psychiatric | 3.69 | 0.071 | 0.89-15.24 | 7.77 | 0.044 | 1.06-57.19 | 3.70 | 0.071 | 0.90-15.28 | 7.91 | 0.042 | 1.08-58.22 |
| Incident unrelated malignant | 0.90 | 0.854 | 0.28-2.83 | 0.91 | 0.867 | 0.29-2.87 | 0.90 | 0.853 | 0.28-2.83 | 0.93 | 0.896 | 0.29-2.94 |
| Incident unrelated somatic | 0.84 | 0.555 | 0.47-1.50 | 0.83 | 0.538 | 0.45-1.52 | 0.87 | 0.547 | 0.56-1.35 | 0.73 | 0.210 | 0.44-1.20 |
| Prevalent diabetes-related | 0.97 | 0.039 | 0.94-1.00 | 0.96 | 0.011 | 0.93-0.99 | 0.97 | 0.038 | 0.94-1.00 | 0.96 | 0.011 | 0.93-0.99 |
| Prevalent unrelated psychiatric | 1.08 | 0.575 | 0.82-1.42 | 1.04 | 0.816 | 0.77-1.39 | 1.08 | 0.583 | 0.82-1.42 | 1.04 | 0.814 | 0.77-1.39 |
| Prevalent unrelated malignant | 1.04 | 0.691 | 0.84-1.30 | 0.96 | 0.736 | 0.76-1.21 | 1.05 | 0.687 | 0.84-1.30 | 0.96 | 0.740 | 0.76-1.21 |
| Prevalent unrelated somatic | 1.03 | 0.182 | 0.99-1.07 | 1.03 | 0.126 | 0.99-1.08 | 1.03 | 0.180 | 0.99-1.07 | 1.03 | 0.118 | 0.99-1.08 |
| New antihypertensive drug started | 0.50 | 0.072 | 0.24-1.06 | 0.38 | 0.057 | 0.14-1.03 | 0.51 | 0.075 | 0.24-1.07 | 0.38 | 0.059 | 0.14-1.04 |
| New lipid-regulating drug started | 0.91 | 0.794 | 0.43-1.91 | 0.73 | 0.534 | 0.27-1.96 | 0.91 | 0.809 | 0.43-1.93 | 0.74 | 0.557 | 0.28-2.00 |
| Aspirin started | - | - | - | - | - | - | - | - | - | - | - | - |
| New unrelated drug started | 0.87 | 0.314 | 0.67-1.14 | 0.97 | 0.859 | 0.72-1.32 | 0.87 | 0.307 | 0.67-1.13 | 0.98 | 0.900 | 0.72-1.33 |
| HbAc (1 %) | 1.34 | 0.000 | 1.28-1.40 | 1.38 | 0.000 | 1.30-1.46 | 1.34 | 0.000 | 1.28-1.40 | 1.38 | 0.000 | 1.30-1.47 |
| Age (10 yrs) | 0.96 | 0.087 | 0.92-1.01 | 0.96 | 0.141 | 0.91-1.01 | 0.96 | 0.088 | 0.92-1.01 | 0.96 | 0.143 | 0.91-1.01 |
| Female | 1.02 | 0.763 | 0.91-1.13 | 1.03 | 0.627 | 0.90-1.18 | 1.02 | 0.760 | 0.91-1.14 | 1.03 | 0.618 | 0.91-1.18 |
| Diabetes duration (10 yrs) | 0.59 | 0.000 | 0.52-0.67 | 0.53 | 0.000 | 0.45-0.62 | 0.59 | 0.000 | 0.52-0.67 | 0.53 | 0.000 | 0.45-0.62 |
| Polypharmacy (≥4 drugs) | 1.00 | 0.974 | 0.89-1.12 | 1.07 | 0.344 | 0.93-1.23 | 1.00 | 0.966 | 0.89-1.12 | 1.07 | 0.355 | 0.93-1.23 |
| Current oral antidiabetic drug user | 0.59 | 0.000 | 0.52-0.68 | 0.61 | 0.000 | 0.51-0.72 | 0.59 | 0.000 | 0.52-0.68 | 0.61 | 0.000 | 0.51-0.72 |
|  |  |  |  |  |  |  |  |  |  |  |  |  |

HR = Hazard ratio, P = P-value, CI = Confidence Interval
